# Metabolic-associated fatty liver disease and liver fibrosis scores as COVID-19 outcome predictors: a machine-learning application

**DOI:** 10.1007/s11739-023-03316-6

**Published:** 2023-06-03

**Authors:** Mirko Zoncapè, Michele Carlin, Manuele Bicego, Andrea Simonetti, Vittoria Ceruti, Anna Mantovani, Francesco Inglese, Giulia Zamboni, Andrea Sartorio, Pietro Minuz, Simone Romano, Ernesto Crisafulli, David Sacerdoti, Cristiano Fava, Andrea Dalbeni

**Affiliations:** 1 Division of General Medicine C, Covid Unit, Department of Medicine, University of Verona, Azienda Ospedaliera Universitaria Integrata of Verona, Verona, Italy; 2 Liver Unit, Department of Medicine, University of Verona, Azienda Ospedaliera Universitaria Integrata of Verona, Verona, Italy; 3 Department of Computer Science, University of Verona, Verona, Italy; 4 Intensive Care Respiratory Unit, Mantua, Italy; 5 Institute of Radiology, Department of Diagnostics and Public Health, University and Azienda Ospedaliera Universitaria Integrata of Verona, Verona, Italy; 6 Division of Emergency Unit and Covid Unit, Department of Medicine, University and Azienda Ospedaliera Universitaria Integrata Verona, Verona, Italy

**Keywords:** MAFLD, Liver steatosis, FIB-4, Machine learning

## Abstract

Patients with COVID-19 and metabolic-dysfunction associated fatty liver disease (MAFLD) appear to be at higher risk for severe manifestations, especially in the youngest decades. Our aim was to examine whether patients with MAFLD and/or with increased liver fibrosis scores (FIB-4) are at risk for severe COVID-19 illness, using a machine learning (ML) model. Six hundred and seventy two patients were enrolled for SARS-CoV-2 pneumonia between February 2020 and May 2021. Steatosis was detected by ultrasound or computed tomography (CT). ML model valuated the risks of both in-hospital death and prolonged hospitalizations (> 28 days), considering MAFLD, blood hepatic profile (HP), and FIB-4 score. 49.6% had MAFLD. The accuracy in predicting in-hospital death was 0.709 for the HP alone and 0.721 for HP + FIB-4; in the 55–75 age subgroup, 0.842/0.855; in the MAFLD subgroup, 0.739/ 0.772; in the MAFLD 55–75 years, 0.825/0.833. Similar results were obtained when considering the accuracy in predicting prolonged hospitalization. In our cohort of COVID-19 patients, the presence of a worse HP and a higher FIB-4 correlated with a higher risk of death and prolonged hospitalization, regardless of the presence of MAFLD. These findings could improve the clinical risk stratification of patients diagnosed with SARS-CoV-2 pneumonia.

**Supplementary Information:**

The online version contains supplementary material available at 10.1007/s11739-023-03316-6.

## Introduction

Coronavirus disease-2019 (COVID-19) is an infectious disease caused by a beta-coronavirus responsible for severe acute respiratory syndrome (SARS-CoV-2), which has rapidly spread worldwide reaching pandemic proportions [1].

The association between hepatic steatosis and obesity/overweight, diabetes and metabolic dysregulation, either alone or in combination, called Non-Alcoholic Fatty Liver Disease (NAFLD) has been recently updated to Liver Disease Associated to Metabolic Dysfunction (MAFLD) by an experts’ consensus [2].

Only few data regarding the prevalence of liver disease, particularly MAFLD, in COVID-19 patients have been published so far. Nevertheless, metabolic patients with fatty liver disease and hepatic involvement seem to be at higher risk for severe COVID-19 manifestations, especially in the youngest decades [3–9].

It has been hypothesized that this link between MAFLD and severity of respiratory manifestations could be explained by the fact that the angiotensin-converting enzyme 2 receptors and the cellular serine protease TMPRSS2 (ACE2/TMPRSS2), used by SARS-CoV-2 [10–12], are more expressed in patients with metabolic-associated hepatic steatosis or steatohepatitis, with a possible facilitation to the entrance of the virus in the cells [13–15], while taking into account that the study by Meijnikman et al. is based on RNA transcriptomic, and not directly on protein levels or ACE2 activity [14].

In fact, the literature data are not completely convincing, and some studies suggest that liver function tests abnormalities could be related to pre-existing abnormalities linked to MAFLD, or could alternatively be consequence of a higher susceptibility of fatty liver cells to SARS-CoV-2 infection, rather than an increased liver uptake of SARS-CoV-2 [16].

Furthermore, abnormalities in liver function tests were observed at the beginning of the pandemic, documenting a strong link between virus infection and liver damage. However, it still remains unclear whether SARS-CoV-2 productively infects and replicates in liver cells or if it has a direct liver-pathogenic effect [17]. Even if an increased risk for severe COVID-19 was documented especially in relation to the admission to the intensive care units (ICU), in some studies no difference in mortality was observed in patients with or without liver steatosis [5, 18].

Considering this link between COVID-19 and liver disease, Fibrosis-4 index (FIB-4), a score used to calculate the risk of severe liver fibrosis in MALFD patients, was recently associated with mortality in COVID-19, regardless of underlying conditions, including liver diseases [19].

Indeed, patients with pre-existing chronic liver diseases in many studies resulted at higher risk of mortality, and FIB-4 at admission was associated with a worse prognosis [19–22].

Several studies have examined prognostic scores in COVID-19 patients to predict either mortality or admission to ICU, but rarely using artificial intelligence (AI) application through machine learning (ML) model, that offers the opportunity to evaluate more subtle relationships between different scores and laboratory markers.

Nowadays, ML algorithms have been developed as clinical prediction tools in different medical fields [23]. In particular, ML has been used to predict SARS-CoV-2 infection and clinical outcome in acute respiratory distress syndrome, post-operative complications, and stroke [24].

However, no studies applied ML in prediction mortality for COVID-19 in MAFLD patients.

The aim of the present study was to identify, using a ML technique, if the presence of MAFLD, and/or an increase in FIB-4, and/or an altered HP, either taken separately or together, could improve the accuracy of prognostic models about death or prolonged hospitalization, in patients affected by COVID-19.

## Materials and methods

### Patients’ cohorts and data collection

This was a bi-centric (Mantua and Verona Hospitals) retrospective longitudinal study, which considered consecutively admitted patients for COVID-19 pneumonia in medical wards with low and medium intensity of care between 28th February 2020 and 1st May 2021.

The following inclusion criteria were considered: a diagnosis of SARS-CoV-2 obtained through nasopharyngeal swabs (a diagnostic method with real‐time reverse‐transcriptase polymerase chain reaction, RT-PCR, was used), age ≥ 18 years, consent to the COVID19-VR register, abdominal ultrasounds (US) or a chest Computed Tomography (CT) scan including hepatic scans.

Patients affected by active hematological diseases, malignant tumors (except for localized melanoma or localized prostate cancer), chronic renal disease (grade IV or end stage renal disease/uremia) [25], hepatic diseases (other than MAFLD), or with recent major events (stroke, myocardial infarction, major surgery) in the last 30 days or during the hospitalization, were excluded.

Various demographics, hematologic, radiological, clinical data of 672 COVID-19 patients were collected for analysis at admission (± 24 h), as well as outcome and therapy: oxygen-therapy at admission, corticosteroids, anticoagulants, hydroxychloroquine, hyperimmune plasma, antiviral therapy, antibiotic therapy, non-invasive ventilation.

The primary endpoints were the prevalence of MAFDL in COVID-19 patients, while secondary endpoints were mortality and prolonged hospitalization (hospitalization for more than 28 days).

The MAFLD subgroup was then categorized according to the new MAFLD criteria.

These consider the presence of hepatic steatosis detected with radiological imaging, associated with:

- Overweight/obesity,
- Or type II diabetes mellitus (DM),
- Or, for lean subjects, other metabolic dysfunctions (at least two of: large waist circumference, hypertension, hypertriglyceridemia, hypercholesterolemia, low HDL-cholesterol, pre-diabetes, insulin-resistance, inflammatory state with PCR > 2 mg/L) [26, 27].

In particular, in our study, hepatic steatosis (as defined in MAFLD definition) was assessed considering the most recent available radiological imaging obtained with abdominal US and/or chest/abdominal CT scans. The CT images were assessed by a single, highly trained radiologist, blinded to the patients’ status, to identify the presence of hepatic steatosis. The diagnosis was based on the attenuation coefficient: the intensity of the gray-color scale in the scans was “converted” in Hounsfield Units (HU). A mean coefficient of 40 HU in 20 cm^2^ areas of the patients’ liver was set as the cut-off to define the presence of hepatic steatosis [28–31]. Moreover, the same radiologist performed a qualitative assessment to identify hepatic steatosis when liver attenuation was sensibly lower than spleen attenuation.

A set of blood tests that we called Hepatic Profile (HP), consisting of alanine aminotransferase, aspartate aminotransferase, gamma-glutamyl transferase, alkaline phosphatase, total bilirubin, direct bilirubin, albumin, was considered to obtain information about liver inflammation and functionality.

Liver fibrosis risk was then estimated calculating FIB-4, using the formula: ${\text{FIB}} - 4 \, = {\text{ Age }}\left( {{\text{years}}} \right) \, \times {\text{ AST }}\left( {U/L} \right)/\left[ {{\text{PLT }}\left( {10^{9} /L} \right) \, \times {\text{ ALT}}^{1/2} \left( {U/L} \right)} \right]$. FIB-4 has been shown to perform better in detecting liver fibrosis than other non-invasive scores, particularly in MAFLD [32, 33]. Nevertheless, in this study FIB-4 was not used with diagnostic purposes but only as a prognostic indicator, in a selected pooulation of COVID-19 patients. For this reason, it was considered as a whole, and the single parameters composing it (in particular platelets and transaminases, which could be affected by the concomitant inflammatory state) were not relevant “per se”.

In our study the whole population was divided in age groups for the statistical analysis: in particular, we focused our survey on the group of hospitalized patients aged between 55 and 75 years, as from our clinical experience in this age group, it was more difficult to predict the outcomes.

Our study was conducted in accordance with the fundamental ethical principles of the Declaration of Helsinki (COVID-19 Register 2636 CESC approved by the Verona and Rovigo Ethical Committee, for both centers).

### Statistical analysis and machine learning

The statistical data analysis was performed using the statistics software Jamovi, Version 1.6—The Jamovi project (2021). Jamovi. (Version 1.6) [Computer Software]. Retrieved from https://www.jamovi.org.

Continuous variables were visually assessed for normality and reported as mean ± standard deviation, whereas comparison of numeric variables was done using either independent sample *T* test or Mann–Whitney *U* test, if not-normally distributed; categorical variables were reported as numbers and percentages, while the comparison was done using Pearson’s chi-squared test or Fisher’s exact test. A *p* value < 0.05 was considered statistically significant.

Since some laboratory data in the COVID-19 sample were missing in some patients, we decided to rely on Artificial Intelligence (AI) application through ML analysis, in an attempt to improve the quality of the data, and include more patients in the analysis that considered both FIB-4 and HP.

The data relating to the presence or absence of MAFLD was established as explained above, as a starting point in our study, while AI was used only to try to recover the missing data of bio-humoral tests.

The Nearest Neighbor imputation was used to fill in missing values, using for each patient data obtained from other patients who showed similar remaining variables in the Hepatic Profile (HP): this algorithm allowed us to recover more than 40% of missing values.

The whole computational analysis is based on a classification analysis: classification represents a particular Pattern Recognition/Machine Learning task in which the goal is to build a model able to predict the category of an unknown object (among a set of pre-specified categories).

More in detail, the whole analysis was accomplished by resorting to the Random Forest classification model (RF; see Supplementary text for more details). Classification accuracy was estimated via a cross-validation strategy, a mechanism which permitted testing the classifier using objects not present in the training set (the objects used to build the model). We employed the Cross Validation variant called 5-Fold Cross Validation (5-FC), in which the available data are divided in 5 random subsets, and then performed 5 classification experiments. The final accuracy is obtained by averaging the accuracies obtained in each of the fivefolds. For all analyzed configurations (age ranges, target, MAFLD) we computed the 5-FC validation classification accuracies of the version of the Random Forest (RF) classifiers, using 100 trees (we used the Matlab routine TreeBagger from the Statistics and Machine Learning toolbox).

Statistical ML model and analysis were conducted by a single highly trained Statistician.

## Results

### General characteristics and results

Between 28th February 2020 and 1st May 2021, 672 patients infected by SARS-CoV-2 admitted in the Mantova and Verona low-medium intensity COVID-19 Units were enrolled in the study. In all patients, the presence of liver steatosis could be assessed either by US and/or CT scan.

Three-hundred-thirty-three patients (49.6%) were classified as MAFLD patients, whose 29.1% were obese, and 30.2% had type 2 diabetes mellitus (DM). Hypertension was the most frequent risk factor (61.1%).

Baseline demographic, comorbidities, therapy, blood tests, and the hepatic scores of patients subdivided according to age are presented in Table 1: statistically significant differences were found between the two groups regarding days of hospitalization and mortality.

**Table 1:** Baseline demographic characteristics, vital parameters, blood tests and medications in the entire cohort and age subgroups

Variable	Total pop. (*n* = 672)	age 55–75 *y* (*n* = 275)	age > 75 *y* (*n* = 291)	*p* value
Male gender, *n* (%)	424 (63.1)	210 (80.8)	153 (52.8)	** < 0.001**
Age, mean ± SD, *y*	70.8 ± 14.8	66.0 ± 6.0	84 ± 5.4	** < 0.001**
Comorbidities, %				
MAFLD	49.6	61.9	39.7	** < 0.001**
Obesity	29.1	42.7	13.8	** < 0.001**
DM	30.2	38.1	31.0	0.361
Hypertension	61.1	65.4	73.4	** < 0.001**
IHD	10.7	5.8	17.2	**0.003**
Atrial fibrillation	6.5	3.1	12.4	** < 0.001**
Concomitant use of drugs, %				
NSAID	2.8	2.3	13.8	0.233
Corticosteroids	2.2	15.0	14.13	0.862
Furosemide	21.0	11.5	37.2	** < 0.001**
ACE-i	23.4	24.6	28.3	0.104
Beta-blockers	30.7	27.7	43.4	** < 0.001**
Antiplatelet agents	23.5	25.4	31.4	**0.025**
DOAC	6.5	3.1	12.4	** < 0.001**
Statins	24.1	26.5	30.7	0.078
Physical parameters, mean ± SD				
SBP mmHg	134.7 ± 23.3	132 ± 21.7	137 ± 24.1	0.076
DBP mmHg	74.2 ± 12.4	74.5 ± 12.1	73.8 ± 12.4	0.585
HR bpm	84.8 ± 18.0	84.4 ± 18.1	85.1 ± 18	0.721
SpO_2_%	93.1 ± 5.4	92.3 ± 5.8	94 ± 4.9	** < 0.001**
BT °C	36.8 ± 1.0	37 ± 1.1	36.7 ± 0.9	**0.005**
Blood tests, mean ± SD				
Hb g/dL	13.0 ± 4.3	13.5 ± 6.2	12.4 ± 2.1	**0.007**
MCV fL	90.2 ± 8.9	90.3 ± 8.2	90.6 ± 9.4	0.819
WBC·10^9^/L	8.8 ± 4.5	8.6 ± 4.4	9.3 ± 4.6	0.060
Neutrophils × 10^9^/L	7.2 ± 4.2	7.3 ± 4.1	7.7 ± 4.5	0.253
Lymphocytes × 10^9^/L	1.1 ± 0.8	1.1 ± 0.7	1.2 ± 0.9	0.057
Platelets × 10^9^/L	228.1 ± 94.5	232 ± 97.2	223 ± 87.3	0.265
CRP mg/L	114.2 ± 119.0	123 ± 99.6	102 ± 85.7	**0.048**
Glycemia mg/dL	142.0 ± 67.0	138 ± 62.7	150 ± 71.3	**0.046**
Creatinine mg/dL	1.3 ± 0.8	1.3 ± 0.8	1.4 ± 0.9	**0.039**
AST IU/L	59.0 ± 151.3	63.8 ± 205	52.6 ± 63.2	0.402
ALT IU/L	44.8 ± 91.5	48.9 ± 122	35.5 ± 36.5	0.086
Total bilirubin mg/dL	0.9 ± 1.9	1.1 ± 4.4	0.8 ± 0.6	0.064
Albumin g/dL	34.0 ± 11.7	32.6 ± 5.8	35 ± 16.8	**0.038**
CPK IU/L	295.9 ± 1013.1	232 ± 449	326 ± 1405	0.352
Ferritin ng/mL	1294.4 ± 2090.5	1473 ± 1766	849 ± 915	** < 0.001**
Blood gas analysis, mean ± SD				
PaO_2_ mmHg	65.9 ± 17.9	66.5 ± 17.3	64.8 ± 19.5	0.290
FiO_2_, %	42.9 ± 20.4	44.7 ± 18.8	41.2 ± 23.0	0.075
Scores				
FIB-4 points	5.5 ± 40.2	3.3 ± 2.2	8.75 ± 60.5	0.153
Days of hospitalization, mean ± SD, days	17.6 ± 14.0	20.6 ± 16.0	15.3 ± 12.9	** < 0.001**
Death, *n* (%)	25.7	18.1	42.4	**0.009**

Numbers in bold represent statistical significance. *ALT* alanine aminotransferase, *AST* aspartate aminotransferase, *BT* body temperature, *CRP* C-reactive protein, *CPK* creatine phosphokinase, *DBP* diastolic blood pressure, *DM* diabetes mellitus, *DOAC* direct oral anticoagulants, *DFIB-4* fibrosis-4, index for liver fibrosis, *FiO*_*2*_ fraction of inspired oxygen, *Hb* hemoglobin, *HR* heart rate, *ICU* intensive care unit, *IHD* ischemic heart disease, *MCV* mean corpuscular volume, *MAFLD* metabolic-dysfunction associated fatty liver disease, *NSAID* non-steroidal anti-inflammatory drugs, *PaO*_*2*_ partial pressure of oxygen in arterial blood, *pop* population, *SBP* systolic blood pressure, *SD* standard deviation, *SpO*_2_ peripheral oxygen saturation, *WBC* white blood cells, *y* years

Any differences were documented between the two centers.

Demographics and clinical characteristics of MAFLD patients and age subgroups are shown in Table 2, and compared with subjects without MAFLD. As expected, metabolic risk factors are more represented in MAFLD subgroup. In particular, when analyzing our MAFLD cohort, we found that cardiovascular and metabolic comorbidities (in particular obesity, ischemic heart disease, peripheral vasculopathy, and cerebro-vascular disease) were related to mortality in univariate analysis. Nevertheless, in multivariate analysis only cerebro-vascular diseases and obesity were related to death, with the evidence of an inverse correlation for the latter (respectively: *p* = 0.04, OR 3.6 (1.05–12.36), and *p* = 0.007, OR 0.44 (0.24–0.8). No statistical evidence was found in the same group of MAFLD when considering the outcome of prolonged hospitalization.

**Table 2:** Baseline demographic characteristics, vital parameters, blood tests and medications in the entire cohort stratified by MAFLD presence and MAFLD age subgroup

Variable	No MAFLD (*n* = 339)	MAFLD (*n* = 333)	*p* value	No MAFLD age 55–75 *y* (*n* = 114)	MAFLD age 55–75 *y* (*n* = 161)	*p* value	No MAFLD age > 75 *y* (*n* = 166)	MAFLD age > 75 *y* (*n* = 125)	*p* value
Male gender, *n* (%)	172 (55.3)	235 (70.6)	** < 0.001**	36 (31.6)	37 (23.0)	0.112	96 (57.8)	52 (45.2)	**0.037**
Age, mean ± SD, *y*	73.7 ± 15.1	68.5 ± 14.0	** < 0.001**	65.9 ± 6.4	66.2 ± 5.7	0.875	84.8 ± 5.5	82.8 ± 4.9	**0.002**
Comorbidities, %									
Obesity	10.6	52.4	** < 0.001**	18.6	60.5	** < 0.001**	4.8	30.5	** < 0.001**
DM	17.7	44.4	** < 0.001**	19.3	47.8	** < 0.001**	17.5	53	** < 0.001**
Hypertension	54.0	68.8	** < 0.001**	42.1	72.7	** < 0.001**	70.5	77.4	0.198
IHD	11.4	13.7	0.401	6.1	11	0.195	15.5	20.8	0.273
Concomitant use of drugs, %									
NSAD	3.5	2.4	0.391	2.7	1.9	0.659	3.6	4.3	0.755
Corticosteroids	15.4	9.9	**0.035**	21.1	8.7	**0.003**	13.9	15.7	0.675
Furosemide	21.9	20.7	0.723	11.4	9.9	0.697	33.1	43.5	0.078
ACE-i	20.9	27.0	0.069	14.9	28.6	**0.008**	28.3	29.6	0.820
Beta-blockers	26.4	34.5	**0.025**	20.2	28.6	0.114	35.5	53.9	**0.002**
Antiplatelet agents	19.3	28.8	**0.005**	13.2	31.1	** < 0.001**	26.5	40	**0.017**
DOAC	7.1	6.0	0.583	2.6	2.5	0.939	11.4	13.9	0.538
Statins	22.5	26.1	0.276	18.4	28.6	0.053	28.9	33	0.460
Physical parameters at admission (mean ± SD)									
SBP mmHg	134 ± 23.0	135 ± 24.1	0.468	131.6 ± 19.4	133.3 ± 24.4	0.633	136.1 ± 24.0	137.5 ± 24.5	0.601
DBP mmHg	73.9 ± 12.3	74.7 ± 12.7	0.369	74.4 ± 11.2	74.9 ± 13.3	0.510	73.6 ± 12.4	74.1 ± 12.4	0.900
HR bpm	84.2 ± 17.0	86.3 ± 19.6	0.218	84.3 ± 18.3	85.0 ± 17.8	0.162	84.2 ± 16.0	86.9 ± 21.4	0.836
SpO_2_%	94.2 ± 4.5	91.9 ± 6.0	** < 0.001**	93.8 ± 4.9	91.2 ± 6.2	** < 0.001**	94.3 ± 4.3	93.3 ± 5.6	0.126
BT °C	36.7 ± 0.9	37.0 ± 1.1	**0.013**	36.8 ± 1.0	37.3 ± 1.2	**0.034**	36.6 ± 0.8	36.8 ± 1.0	0.435
Laboratory blood tests at admission, (mean ± SD)									
Hb g/dL	12.9 ± 2.0	12.9 ± 2.1	0.658	13.3 ± 1.9	13.7 ± 8.1	0.532	12.5 ± 2.0	12.3 ± 2.1	0.508
WBC × 10^9^/L	9.1 ± 4.5	8.4 ± 4.2	0.087	8.8 ± 4.1	8.3 ± 4.6	0.198	9.3 ± 4.6	9.2 ± 4.2	0.974
Neutrophils × 10^9^/L	7.5 ± 4.3	6.9 ± 4.0	0.073	7.6 ± 3.9	7.0 ± 4.1	0.132	7.6 ± 4.4	7.7 ± 4.2	0.832
Lymphocytes × 10^9^/L	1.1 ± 0.8	1.2 ± 0.8	0.870	1.0 ± 0.6	1.1 ± 0.8	0.527	1.1 ± 0.8	1.3 ± 0.9	0.683
Platelets ·10^9^/L	230.0 ± 88.6	224.0 ± 98.9	0.159	231.5 ± 91.4	231.4 ± 103.1	0.686	229.2 ± 83.8	213.4 ± 90.8	0.077
CRP mg/L	102.0 ± 8.80	129.0 ± 1,4	**0.006**	107.8 ± 76.2	137.2 ± 188.2	0.377	96.5 ± 78.5	111.2 ± 95.5	0.154
Glycemia mg/dL	141.0 ± 64.1	144.0 ± 70.7	0.815	143.4 ± 72.3	134.1 ± 53.6	0.856	142.5 ± 59.0	163.0 ± 87.2	**0.042**
Creatinine mg/dL	1.3 ± 0.8	1.3 ± 0.9	0.285	1.1 ± 0.35	1.4 ± 1.1	0.490	1.46 ± 1.0	1.3 ± 0.6	0.382
AST IU/L	67.7 ± 209.0	50.3 ± 42.3	0.680	74.7 ± 30.5	55.2 ± 51.7	0.537	56.8 ± 77.0	46.4 ± 32.8	0.253
ALT IU/L	46.7 ± 125.0	43.1 ± 35.8	**0.002**	54.4 ± 18.4	44.8 ± 34.2	**0.026**	35.3 ± 37.2	35.8 ± 35.6	0.582
Total bilirubin, mg/dL	0.7 ± 1.1	1.1 ± 2.7	**0.002**	0.8 ± 1.7	1.5 ± 4.0	**0.004**	0.7 ± 0.6	0.8 ± 0.4	0.181
Albumin g/dL	48.0 ± 5.00	54.0 ± 5.2	0.081	32.7 ± 5.6	32.6 ± 6.0	0.659	36.3 ± 21.2	33.1 ± 4.9	**0.011**
CPK IU/L	271.0 ± 1312.0	321.0 ± 620.0	0.125	148.8 ± 278.3	296.6 ± 538.0	**0.002**	380.5 ± 1812.9	246.8 ± 347.2	0.617
Ferritin ng/mL	1356.0 ± 2703.0	1248.0 ± 1383.0	0.278	1584.5 ± 2094.3	1382.4 ± 1528.3	0.815	872.3 ± 914.0	807.0 ± 930.2	0.282
Blood gas analysis at admission, mean ± SD									
PaO_2_ mmHg	66.4 ± 18.4	65.6 ± 17.7	0.366	68.5 ± 20.0	64.7 ± 15.0	0.093	65.3 ± 17.1	64.9 ± 22.9	0.238
FiO_2_%	40.7 ± 21.4	44.3 ± 19.4	**0.003**	43.9 ± 19.7	44.9 ± 18.7	0.560	38.5 ± 22.9	43.6 ± 22.3	**0.013**
P/F ratio	203.0 ± 94.4	189.0 ± 105.0	**0.037**	188.0 ± 80.7	185.8 ± 112.6	0.278	216.4 ± 102.8	198.6 ± 104.1	0.178
Scores									
FIB-4, mean ± SD, points	6.5 ± 54.0	4.5 ± 17.5	**0.038**	3.1 ± 4.2	3.6 ± 5.4	0.889	9.9 ± 74.7	7.0 ± 8.3	0.956
Days of hospitalization, mean ± SD, days	16.4 ± 11.9	18.8 ± 15.5	0.087	18.8 ± 13.8	21.8 ± 16.9	0.094	14.9 ± 10.8	16.0 ± 15.7	0.489
Death, *n* (%)	28.0	25.0	0.393	14.9	18.8	0.406	41.6	44.3	0.643

Numbers in bold represent statistical significance. *ALT* alanine aminotransferase, *AST* aspartate aminotransferase, *BT* body temperature, *CRP* C-reactive protein, *CPK* creatine phosphokinase, *DBP* diastolic blood pressure, *DM* diabetes mellitus, *FIB-4* fibrosis-4, index for liver fibrosis, *FiO*_2_ fraction of inspired oxygen, *Hb* hemoglobin, *HR* heart rate, *ICU* intensive care unit, *IHD* ischemic heart disease, *MCV* mean corpuscular volume, *MAFLD* metabolic-dysfunction associated fatty liver disease, *PaO*_2_ partial pressure of oxygen in arterial blood, *SBP* systolic blood pressure, *SD* standard deviation, *SpO*_2_ peripheral oxygen saturation, *WBC* white blood cells, *y* years

Concerning the SpO2 in MAFLD cohort, no correlations with mortality and prolonged hospitalization were found for SpO2 ad admission in the MAFLD cohort (respectively: *p* = 0.450, *p* = 0.140).

Even if the two subgroups differed for many characteristics, there were no statistically significant differences in mortality and prolonged hospitalization in subjects with MAFLD as compared with those without MAFLD (see Table 2).

As for FIB-4, there were no significant differences between the younger (55–75 years) versus the older (> 75 years) cohorts. Moreover, there were no significant differences between the MAFLD and the non-MAFLD groups, only based on FIB-4.

### Machine learning analysis

**Fig. 1:**
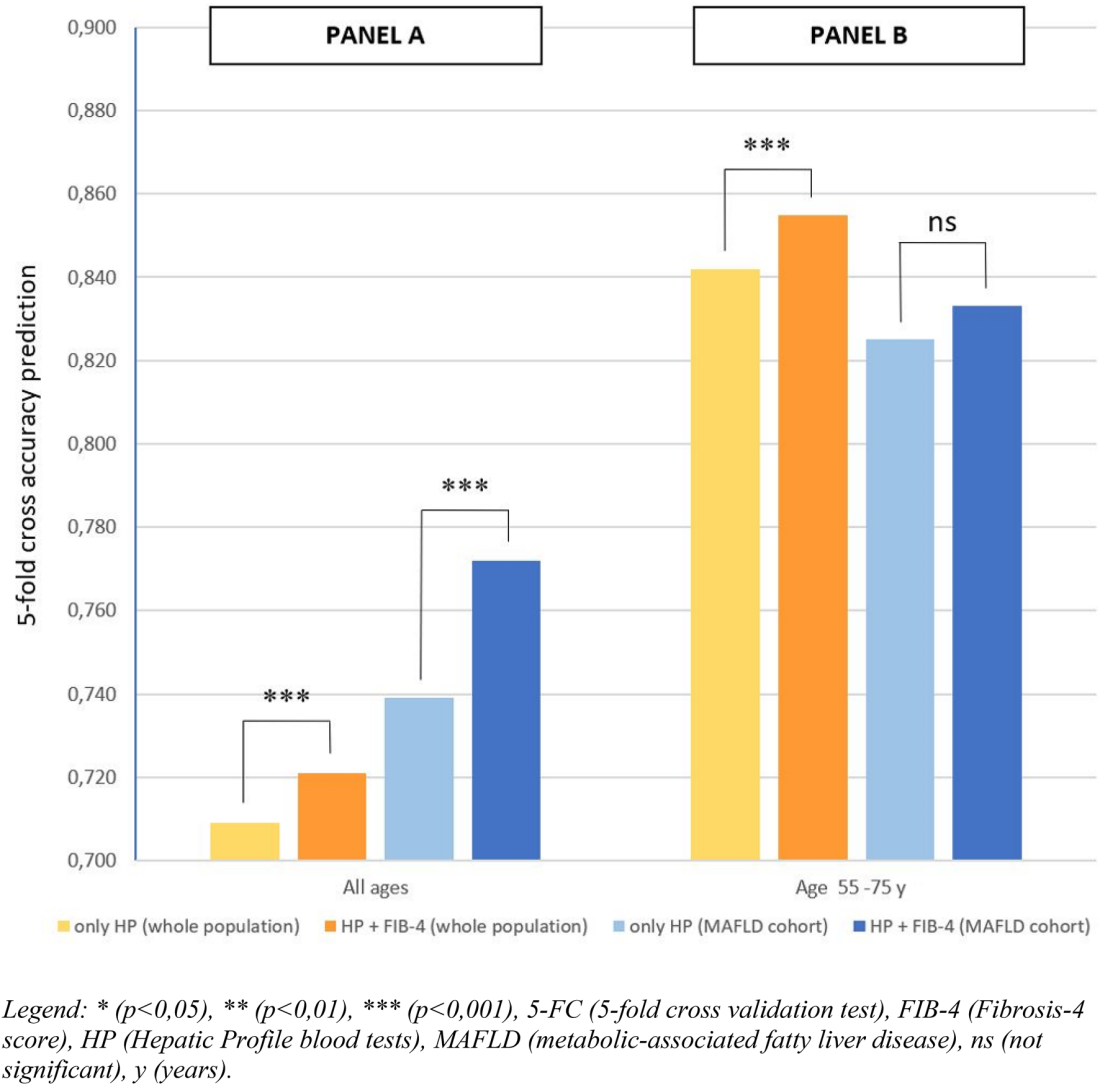
Bar graph showing the accuracy in death prediction with the fivefold cross validation test in our COVID-19 population, considering different sample, considering different subgroups (“Panel A” describe the all-ages cohort, while “Panel B” describe 55–75 age group) comparing the use of HP alone or the combined use of HP and FIB-4. *p < 0.05, **p < 0.01, ***p < 0.001, *5-FC* fivefold cross validation test, *FIB-4* Fibrosis-4 score, *HP* Hepatic profile blood tests, *MAFLD* metabolic-associated fatty liver disease, *ns* not significant, *y* years

**Fig. 2:**
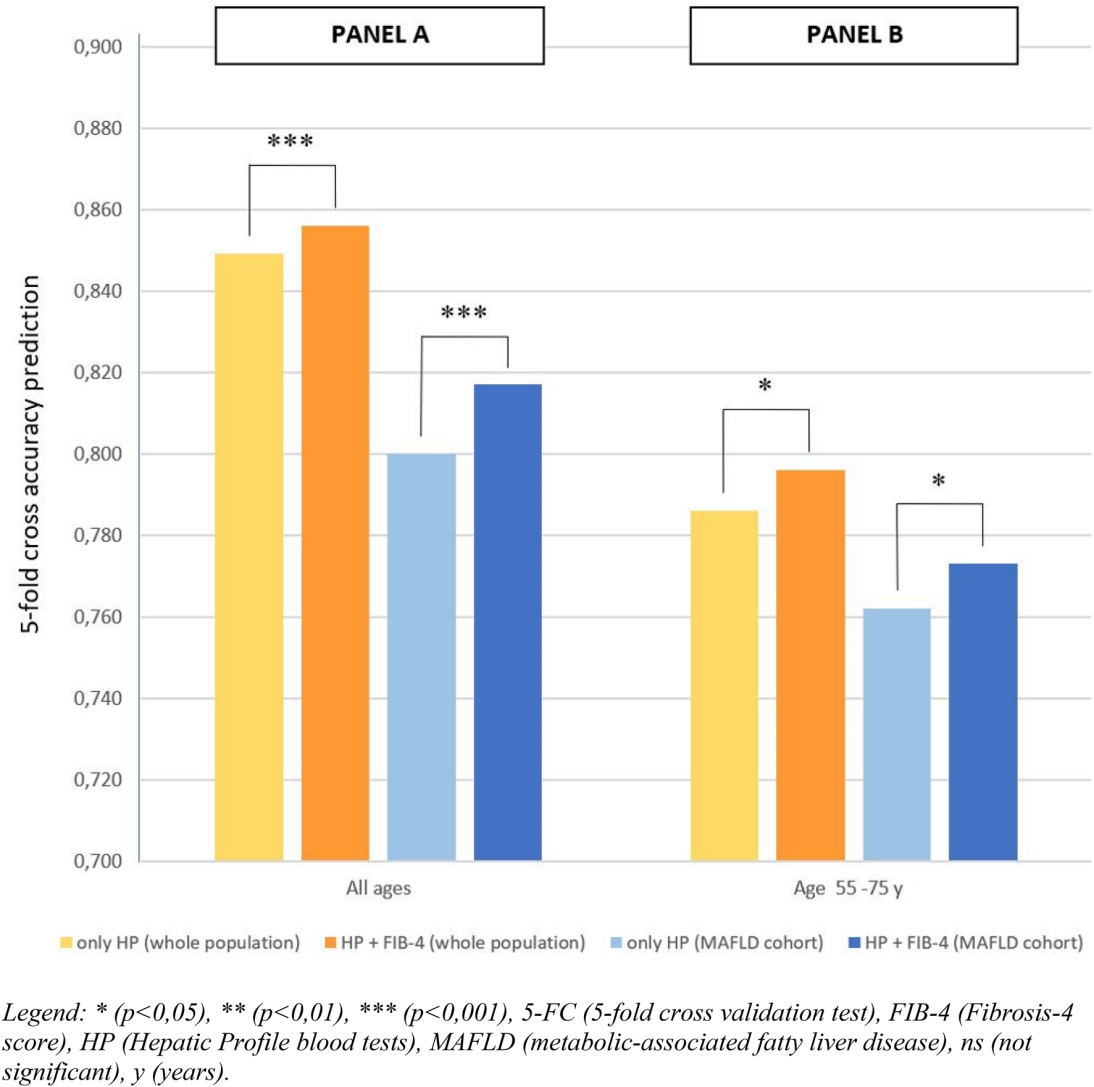
Bar graph showing the accuracy in prolonged hospitalization (> 28 days) prediction with the fivefold cross validation test in our COVID-19 sample, considering different subgroups (“Panel A” describe the all-ages cohort, while “Panel B” describe 55–75 age group) and comparing the use of HP alone or the combined use of HP and FIB-4. *p < 0.05, **p < 0.01, ***p < 0.001, *5-FC* fivefold cross validation test, *FIB-4* Fibrosis-4 score, *HP* Hepatic profile blood tests, *MAFLD* metabolic-associated fatty liver disease, *ns* not significant, *y* years

ML results are reported in bar graphs showing the prediction accuracy in the different cohorts (Figs. 1 and 2). Considering the whole COVID-19 population through the HP, using ML analysis, we observed a more accurate prediction for both death (accuracy of 0.709 for all ages and 0.842 for the subgroup 55–75-years) and prolonged hospitalization (accuracy of 0.849 for the whole population and 0.786 for the 55–75 years subgroup, considering the HP), as seen in Table 1S (in supplementary data). Moreover, in the whole COVID-19 sample, the combined FIB-4 and HP predicted mortality (accuracies of 0.721 in all-ages group, and 0.855 in the younger subgroup) and prolonged hospitalization (accuracies of 0.856 in the entire sample, and accuracy of 0.796 in the 55–75-years), lead to higher accuracies than the single indices separately (Figs. 1 and 2). Similar results were obtained when applying the ML with FIB-4 and HP specifically to the MAFLD subgroup, as shown in Table 1S.

In the MAFLD cohort we observed similar results when we considered FIB-4 and HP together, compared to HP alone, both in the whole group and in the 55–75 years subgroup with the exception of the accuracy in the prediction of death in the 55–75 age subgroup (see Figs. 1 and 2: “Panels A” describe the all-ages cohort, while “Panels B” describe 55–75 age group).

We also performed the statistical analyses adding the FIB-4 index in the non-MAFLD patients, but there was no improvement in accuracy (see Table 1S in supplementary data).

## Discussion

Recently, many articles looked for prognostic scores that could predict major outcomes such as death or hospitalization in COVID-19 patients, in particular when MAFLD is present.

A study conducted on 256 patients with unknow liver disease between February and May 2020, had shown that FIB-4 score had a good prognostic power, well correlating with the need for intensive support and mechanical ventilation as well as with 30-day mortality, when associated with particular comorbidities (such as obesity, DM and known history of respiratory diseases) [20].

In another study by Ibáñez-Samaniego et al. on 160 COVID-19 patients between 35 and 65 years old, a FIB-4 above 2.67 showed a prognostic role, being associated with poor outcomes: patients were more likely to require mechanical ventilation or intensive care support [34]. This study was conducted in patients with a history of COVID-19 but without accurate information about MAFLD presence, although the authors agreed that the prevalence of liver fibrosis (≥ stage 2) is mostly attributed to MAFLD in the general population. Li et al. in 2020 conducted a study in which FIB-4 score was calculated in 202 hospitalized patients with COVID-19: the authors noticed that FIB-4 score elevation could be multifactorial and showed that it was associated with mortality [35]. Similarly, Park et al. demonstrated that FIB-4 correlates with mortality in COVID-19 patients, suggesting its use as a useful predictive marker [36]. Similar results were obtained by Sterling et al., valuating FIB-4 score in 256 hospitalized patients: a higher FIB-4 score correlated with a more frequent need of mechanical ventilation and intensive care support [20].

We decided to investigate the prognostic value of a diagnosis of MAFLD either alone or in combination with FIB-4 and/or HP. To optimize the quantity and reliability of our retrospective data, we used an AI application through the ML method, and selected those tests and scores that are easy to obtain (blood tests and FIB-4). Especially in recent years, several studies based on ML have proved useful to improve the predictive reliability of the data under examination. In a study conducted in 2021 in 3,058 patients (13.8% of them with a confirmed diagnosis of COVID-19 pneumonia), authors developed a machine learning model to detect COVID‐19 and other subtypes of pneumonia: the ML application was successful to correctly predict SARS‐CoV‐2 infection using blood tests and chest radiographs [37].

Even in our dataset, using ML, elderly patients (over 75 years of age) had higher mortality rates and poor response to supportive care, while younger patients, especially under 55 years of age had good prognosis with longer survival, shorter hospitalizations and better therapeutic responses.

However, poor outcomes remain partly unexplained in the intermediate-age population (between 55 and 75 years), with prolonged hospitalizations and high mortality rates.

Our results show that MAFLD alone in COVID-19 patients cannot predict mortality or prolonged hospitalization.

This is in agreement with the observations of Mushtaq et al. in which NAFLD was a predictor of mild or moderate liver injury in hospitalized patients with COVID-19, but it was not an independent predictor of mortality or disease progression [5], and with the study of Lopez-Mendez et al., in which the prevalence of liver steatosis and advanced fibrosis (determined by FIB-4) was high in COVID-19 patients and it was not associated with clinical outcomes [38].

Also, in the study by Campos-Murguía et al., the authors concluded that, considering the presence of MAFLD alone, there was no statistical difference in worse outcomes, but fibrosis, was associated with an increased risk of mechanical ventilation, development of acute kidney injury and higher mortality in COVID-19 patients [39].

More recently, a systematic review on 8736 hospitalized patients with COVID-19, suggested that liver fibrosis scores, including the FIB-4 were significantly associated with the increased risk of severe COVID-19, mechanical ventilation, and mortality [22].

Even if the presence of MAFLD by itself cannot predict mortality in our sample, by adding the FIB-4 to the prediction model, sensitivity and specificity increased significantly. Moreover, the combination of FIB-4 score and the HP greatly improves sensitivity and specificity in predicting mortality in different subgroups (with and without MAFLD and with different ages) [7, 34].

Different studies suggested that advanced liver fibrosis may increase the risk of developing an enhanced inflammatory response after SARS-CoV-2 infection, leading to severe COVID-19.

On the other hand, both the FIB-4 and the HP can be altered not for the presence of significant chronic liver fibrosis or inflammation/dysfunction, but for an acute insult to the liver by the virus or the drugs used even before hospitalization.

Our study has strengths and limitations. Among the strengths, is the relatively large sample size with specific information about MAFLD or FIB-4, and the application of ML both to recover data and to estimate prognostic models.

Another important strength is the availability of liver imaging for all the patients, making it possible to obtain information about the presence or absence of MAFLD in the analyzed COVID-19 population. However, our study is also characterized by some limitations. First of all, there are epidemiological differences between SARS-CoV-2 infection and MAFLD prevalence. Second, this was a retrospective study with prospectively collected data, meaning that we had some missing values, retrieved by ML to get as close as possible to the real ones. Moreover, the diagnosis of MAFLD is based on anamnestic factors and the presence of hepatic steatosis: the steatosis of the liver is based on different radiological imaging (CT or US scans) and especially US is an operator-dependent radiological method, that could potentially lead to misclassification. Different radiological methodscan lead to an interpretative bias, which we have tried to overcome, ensuring that the radiological techniques were performed by an expert operator, blind to the patients. Furthermore, the scores and exams we used cannot discriminate between chronic fibrosis/hepatocellular dysfunction and an acute injury. The addition of fibroscan or any other type of hepatic elastography (e.g., 2D-ShearWave Elastometry) could have added an aid to this aim, although they are not so easy to perform in COVID-19 patients.

## Conclusions

The association of HP tests with FIB-4 score in COVID-19 subjects can give a more accurate prediction of adverse outcomes (death or prolonged hospitalization), regardless of the age subgroup or MAFLD presence. These results could improve the clinical risk stratification at hospital admission of patients diagnosed with SARS-CoV-2 pneumonia. This also applies to the age group between 55 and 75 years, which showed the worst outcomes despite the use of maximal care in our population. Furthermore, this may pave the way for finding a better prognostic algorithm in subjects with MAFLD. On the contrary, no significant correlations were found in prediction of outcomes for the non-MAFLD cohort.

## Supplementary Information

Below is the link to the electronic supplementary material.

Supplementary file1 (DOCX 58 KB)

Supplementary file2 (DOCX 17 KB)

## Data Availability

Not applicable.
