# Supplementary material for: Metabolic-associated fatty liver disease and liver fibrosis scores as COVID-19 outcome predictors: a machine-learning application
Source: Intern Emerg Med. 2023 Jun 3;18(7):2063–73. doi: 10.1007/s11739-023-03316-6 (PMC10238243; doi:10.1007/s11739-023-03316-6)
Supplement: Supplementary file 1 — Supplementary file1 (DOCX 58 KB) [file 11739_2023_3316_MOESM1_ESM.docx]

**Metabolic-associated fatty liver disease and liver fibrosis scores as COVID-19 outcome predictors: a machine-learning application.**

**Supplementary** **MATERIALS AND METHODS**

**Machine Learning**

The whole computational analysis is based on a classification analysis: classification represents a particular Pattern Recognition/Machine Learning task in which the goal is to build a model able to predict the category of an unknown object (among a set of pre-specified categories). The model is typically learned using the “learning from examples paradigm”, i.e. using a set of objects sampled from the problem for which the label is known (the so-called training set) [40, 41]. Machine Learning and Pattern Recognition techniques are increasingly used in medicine, often providing not obvious alternative perspectives on data, which are not easily detected using classical statistical tests [42].

More in detail, the whole analysis was accomplished by resorting to two different classification models: NIR (No Information Rate) and RF (Random Forest). The NIR (No Information Rate) value is a common measure used to establish a sort of baseline result: it represents the accuracy of a naive classifier that assigns all objects to the most frequent class in the training set. Random Forest is an ensemble method, meaning that it is made up of a large number of small estimators, each producing their own predictions, and that it combines them to produce a more accurate prediction [43].

Statistical machine learning model and analysis were conducted by a single highly-trained Statistician. The classification accuracy was estimated via a cross-validation strategy, i.e. a mechanism which permitted testing the classifier using objects not present in the training set (the objects used to build the model). Cross-validation permitted a fair evaluation of the generalization capability of the model, i.e. its capability in classifying also objects not present in the training set [41].

In particular we employed the Cross Validation variant called 5-Fold Cross Validation (5-FC), in which the available data is divided in 5 random subsets (called folds), and then performed 5 classification experiments: in the first, we train the classifier using all folds except the first, which is then used for testing it. In the second step, we train the classifier with all folds except the second, which is then used for testing. The procedure is repeated until all folds have been used for testing. The final accuracy is obtained by averaging the accuracies obtained in each of the 5 folds. To compute the accuracy, we compared the labels predicted by the classifier with the true labels of the objects: the accuracy is the number of correctly assigned objects with respect to the total number of objects. In order to increase the strength of the whole procedure, and to remove the variability possibly due to the random creation of the 5 folds, the whole procedure has been repeated 20 times, thus obtaining 20 different accuracies for all possible experiments. In the tables we reported the averaged accuracies, averaged over the 20 repetitions (see Table 1S – supplementary data). With this new protocol we can directly compare with a statistical test the 20 accuracies obtained with the HP configuration with the corresponding 20 obtained with the HP + FIB-4 configuration. In the tables, for every experiment, we also report the p-value corresponding to such comparisons (see Table 1S – supplementary data).

Two statistical tests have been used. T-test, in which the null hypothesis is that the differences between the two sets of accuracies come from a normal distribution with mean equal to zero and unknown variance. A low p-value indicates that the accuracies of HP and those of HP + FIB-4 are different with a statistical significance.

Wilcoxon signed rank test, a non-parametric test, preferable when the number of samples is low. We used the paired version, in which the null hypothesis is that the difference between the two sets of accuracies come from a distribution with zero median (as before, a low p-value indicates that the accuracies of HP and those of HP + FIB-4 are different with a statistical significance).

In particular, we started from the dataset and performed the following steps:

- We removed the patients whose targets (Death/hospitalization), FIB-4, MAFLD or age were missing.
- We described each patient with a set of liver profile variables which we called Hepatic Profile (HP), comprehensive of alanine aminotransferase (AST), aspartate aminotransferase (ALT), gamma-glutamyltransferase (GGT), alkaline phosphatase (ALP), total bilirubin, direct bilirubin and albumin. Missing values were imputed using the Nearest Neighbour imputation (44): in this scheme a missing parameter is replaced with that of the subject with the most similar profile (in this case the most similar hepatic profile).
- We performed a non-linear scaling of the data using the log2 function (non-linear normalization of data has shown to be very useful for classification) [45].
- For all analysed configurations (age ranges, target, MAFLD) we computed the 5-FC validation classification accuracies of the version of the Random Forest (RF) classifiers, using 100 trees (we used the Matlab routine TreeBagger from the Statistics and Machine Learning toolbox).
