# Supplementary material for: Metabolic-associated fatty liver disease and liver fibrosis scores as COVID-19 outcome predictors: a machine-learning application
Source: Intern Emerg Med. 2023 Jun 3;18(7):2063–73. doi: 10.1007/s11739-023-03316-6 (PMC10238243; doi:10.1007/s11739-023-03316-6)
Supplement: Supplementary file 2 — Supplementary file2 (DOCX 17 KB) [file 11739_2023_3316_MOESM2_ESM.docx]

***Table 1S: Statistics of dataset and classification accuracies resulting from machine learning and pattern recognition techniques in total population, in MAFLD cohort and in non-MAFLD cohort.***

| **First analysis: whole population** | | | | | | | |
| --- | --- | --- | --- | --- | --- | --- | --- |
| **Classification accuracies** | | | | | | | |
| **OUTCOME: death** | | | | **hospitalization >28 days** | | | |
|  | HP | HP+FIB-4 | p-value |  | HP | HP+FIB-4 | p-value |
| whole population | 0.709 | 0.721 | **<0.001** | whole population | 0.849 | 0.856 | **<0.001** |
| age 55-75 years | 0.842 | 0.855 | **<0.001** | age 55-75 years | 0.786 | 0.796 | **<0.05** |

| **Second analysis: Only MAFLD subjects** | | | | | | | |
| --- | --- | --- | --- | --- | --- | --- | --- |
| **Classification accuracies** | | | | | | | |
| **OUTCOME: death** | | | | **hospitalization >28 days** | | | |
|  | HP | HP+FIB-4 | p-value |  | HP | HP+FIB-4 | p-value |
| whole population | 0.739 | 0.772 | **<0.001** | whole population | 0.800 | 0.817 | **<0.001** |
| age 55-75 years | 0.825 | 0.833 | 0.076 | age 55-75 years | 0.762 | 0.773 | **<0.05** |

| **Third analysis: non-MAFLD subjects** | | | | | | | |
| --- | --- | --- | --- | --- | --- | --- | --- |
| **Classification accuracies** | | | | | | | |
| **OUTCOME: death** | | | | **hospitalization >28 days** | | | |
|  | HP | HP+FIB-4 | p-value |  | HP | HP+FIB-4 | p-value |
| whole population | 0.664 | 0.663 | 0.590 | whole population | 0.865 | 0.865 | 0.920 |
| age 55-75 years | 0.825 | 0.823 | 0.380 | age 55-75 years | 0.788 | 0.795 | 0.062 |

*Legend: FIB-4 (Fibrosis-4 index), HP (Hepatic Profile blood tests).*
